# An NGS-based approach for the identification of sex-specific markers in snakehead (*Channa argus*)

**DOI:** 10.18632/oncotarget.21924

**Published:** 2017-10-19

**Authors:** Mi Ou, Cheng Yang, Qing Luo, Rong Huang, Ai-Di Zhang, Lan-Jie Liao, Yong-Ming Li, Li-Bo He, Zuo-Yan Zhu, Kun-Ci Chen, Ya-Ping Wang

**Affiliations:** ^1^ State Key Laboratory of Freshwater Ecology and Biotechnology, Institute of Hydrobiology, Chinese Academy of Sciences, Wuhan 430072, China; ^2^ University of Chinese Academy of Sciences, Beijing 100049, China; ^3^ Pearl River Fisheries Research Institute, Chinese Academy of Fishery Sciences, Guangzhou 510380, China

**Keywords:** next generation sequencing, sex-specific molecular markers, snakehead

## Abstract

We described a next generation sequencing (NGS)-based approach to identify sex-specific markers and subsequently determine whether a species has male or female heterogamety. To test the accuracy of this technique, we examined the snakehead (*Channa argus*), which is economically important freshwater fish in China. Males grow faster than females, and there is significant interest in developing methods to skew breeding towards all-males to increase biomass yields. NGS was conducted on DNAs of individual female and male, the male reads were spitted into 60 bp K-mers and aligned to the female reference genome assembled by female reads, unaligned male K-mers-60 were kept in next filter process. Meanwhile, DNA sample of 48 females was pooled and sequenced, this data was further used to filter out the previous unaligned male K-mers-60. Hence, numbers of candidate Y chromosome-specific sequences were screened out, their sex-specificity were validated in wild snakeheads through PCR amplification. Finally, three Y chromosome-specific fragments (Contig-275834, Contig-359642, and Contig-418354) were identified, and specific primers were obtained to distinguish the sex of snakehead. Additionally, a pair of primers of Contig-275834 (275834X/Y-F and 275834X/Y-R) was exploited to distinguish XX females, XY males, and YY super-males, whose amplification products of different lengths were produced for different sexes. Therefore, our work demonstrated the ability of NGS data in identification of sex-specific markers, and the pipeline adopted in our study could be applied in any species of sex differentiation. Furthermore, the sex-specific markers have tremendous potential for improving the efficiency of all-male breeding practices in snakehead.

## INTRODUCTION

The mechanisms of sex determination in animals are remarkably diverse. As primitive vertebrates, it commonly has sex determination systems with either XX/XY (male heterogamety), or ZZ/ZW (female heterogamety) in fish [1]. Growth is one of the most valuable economic traits for fish genetic improvement. Because some fish species display different growth rates and body sizes for different sexes, so all-female or all-male population production has significant economic implications in aquaculture [2, 3, 4]. It is important and meaningful to search for a convenient and forthright method to identify the genetic sex of fish in aquaculture. Identifying the sex chromosome system is typically done using one of three techniques: cytogenetic approaches that visualize heteromorphic sex chromosomes [5]; test cross experiments involving sex-reversed fish [6]; or the identification of sex-specific molecular markers [7]. However, no heteromorphic sex chromosomes have been found in most of fish species. Even in fish with heteromorphic sex chromosomes, it is difficult to discriminate through sex chromosome morphology, since the differentiation and divergent degree of sex chromosome is very low [8]. Similarly, hormonal sex reversal and test crosses are laborious and time consuming, thus the identification of sex-specific markers holds the most promise as an approach to identify sex chromosomes.

The restriction fragment length polymorphism (RFLP) approach was applied to Chinook Salmon (*Oncorhynchus tshawytscha*) in 1991, and a DNA fragment from the Y chromosome was discovered that could be used for sex genotyping [9], it was the first report that molecular marker could be used to screen sex-specific loci in fish. Simple Sequence Repeats (SSRs) and Amplified Fragment Length Polymorphism (AFLP) are the second generation of molecular markers, and SSRs have been applied to identify sex-specific molecular markers in several fish [3, 10, 11, 12]. Meanwhile, many sex-specific loci have been successfully identified by AFLP technique in some important fish [13, 14, 15, 16, 17]. Recently, Restriction-Site Associated DNA (RAD) sequencing has been proven to be a powerful technique for uncovering sex-specific molecular markers [18], which bases on the third generation of molecular markers-Single Nucleotide Polymorphisms (SNPs) and takes advantage of data from NGS. Combining with high-density linkage maps and QTL analyses, RAD sequencing have identified many sex-associated loci in fish [19, 20, 21, 22, 23].

Snakehead (*Channa argus*), a freshwater fish of the *Channidae* family, is an economically important freshwater fish native to East Asia. It is regarded as valuable fish because of its high protein content, significant anti-hypoxia capacity and even curative function in traditional Chinese medicine [24]. Total production of snakeheads reached 510,340 tonnes in 2014, ranking ninth in the production of all freshwater fish species in China [25]. Field surveys and aquaculture practices have demonstrated that there is significant sexual dimorphism in the growth rates and body sizes of males versus females. Specifically, males grow faster than females, resulting in a twofold size difference after 2-3 years of culture [26]. Therefore, it would be useful to exploit sex manipulation biotechnologies to develop breeding strategies that preferentially produce all-male snakeheads. Karyotyping indicates that snakehead is diploid (2n=48), but heteromorphic sex chromosomes have not been identified [27]. Meiotic gynogenetic snakeheads are all-female, suggesting a sex determination system with XX/XY (private communication). In order to produce all-male snakeheads, we should first obtain YY super-males through hormonal sex reversal and test crosses, and then mate YY super-male with normal XX female [28, 29]. In traditional all-males breeding, people use test crosses and count the sex ratio of the offspring to determine the sex of the fish. However, test crosses are laborious and time consuming, and snakeheads need 2 years to reach sexual maturity. Therefore, an approach to accurately and rapidly identify YY super-males without test crosses is urgently required for the artificial sex control of this species. Some researchers have tried to identify sex-specific molecular markers in snakehead. Liu et al. [26] utilized SSRs to screen sex-specific loci from a snakehead family, and one locus was found to be female-specific under certain conditions. Furthermore, AFLP was also employed to screen sex-specific markers in snakehead, and one female-specific AFLP fragment was identified in three breeding families that derived from Foshan City (Guangdong Province) [30]. However, this female-specific AFLP fragment could not be used in other population. Thus, further studies are needed to develop stable and universal sex-specific markers in snakehead.

The traditional techniques to identify sex-specific markers, such as RFLP, SSRs and AFLP, all need high cost, heavy work, and long time, resulting in the slow progress in the study of sex chromosomes and sex determining genes. RAD sequencing discovers SNPs in different sexes based on sequencing the DNA flanking a specific restriction site. In fact, additional sex-specific genomic differences are more than SNPs existing in the genome, such as insertions and deletions (In/Dels), especially in X and Y chromosome, or W and Z chromosome. In this study, we focused on method for the identification of In/Dels in sex chromosomes of snakehead. We utilized NGS data from one male, one female and one female pool of 48 snakeheads, and applied bioinformatics methods to pinpoint genetic differences in sex chromosomes. We aimed to develop a workflow to identify sex-specific molecular markers in snakehead without test crosses and establish a simple PCR genotyping method to distinguish XX females, XY males, and YY super-males, thus improving the efficiency of all-male breeding practices in snakehead.

## RESULTS

### Assembly of the female genome

**Figure 1:**
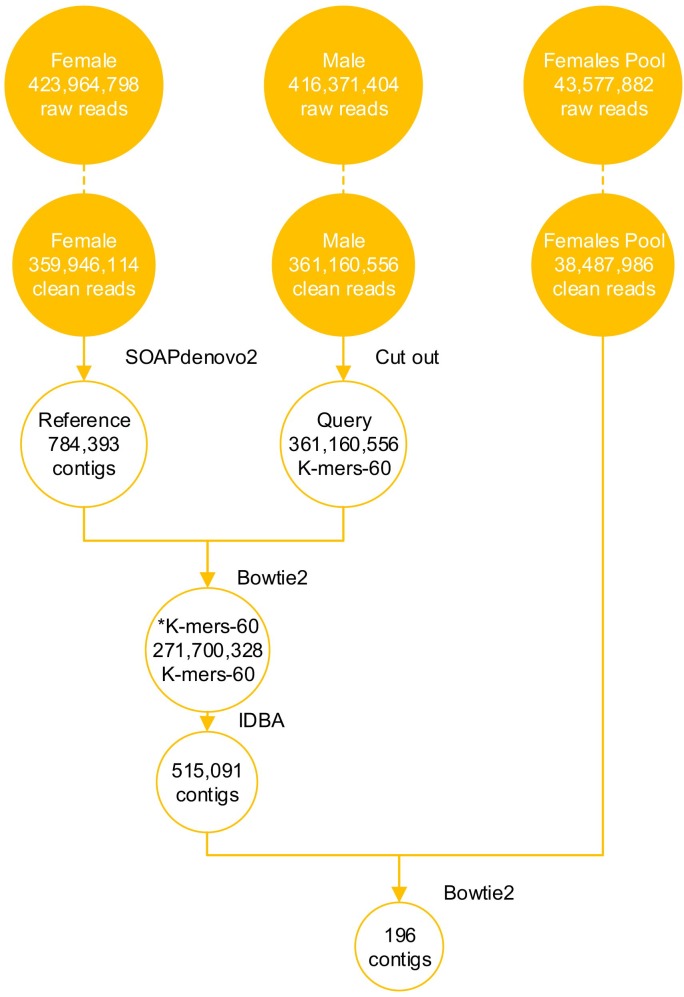
NGS data of snakeheads and analysis data during screening the sex-specific molecular markers DNAs of M1, F1, F’-Mix were used for DNA sequencing. Libraries were separately run in three lane of an Illumina HiSeq 2000, using 150 base paired-end reads. The raw reads were filtered, and obtained clean reads. 196 candidate Y chromosome-specific fragments were screened from the NGS data through bioinformatics analysis, such as genome assembly and alignments.

The filtered data from F1 library came to 53.99 Gb and contained 359,946,114 paired-end (PE) reads (Figure 1), covering ~ 40.27 fold of the reported calculated whole genome [31]. A final genome assembly of 646 Mb in length was obtained, the female genome assembly had 784,393 contigs with an N50 length greater than 3.9 Kb and 507,119 scaffolds with an N50 length greater than 37.0 Kb (Table 1).

**Table 1:** Summary results of the female snakehead assembly by SOAPdenovo2

Type	Contig		Scaffold	
	Size (bp)	Num.	Size (bp)	Num.
N50	3,969	44,292	37,041	4,970
N60	2,964	63,251	27,287	7,109
N70	2,044	89,571	18,162	10,139
N80	1,116	131,807	8,993	15,326
N90	205	269,680	677	41,744
Longest	58,824	1	367,208	1
Total	650,932,685	784,393	677,615,821	507,119

### Acquisition of candidate Y chromosome-specific fragments

**Figure 2:**
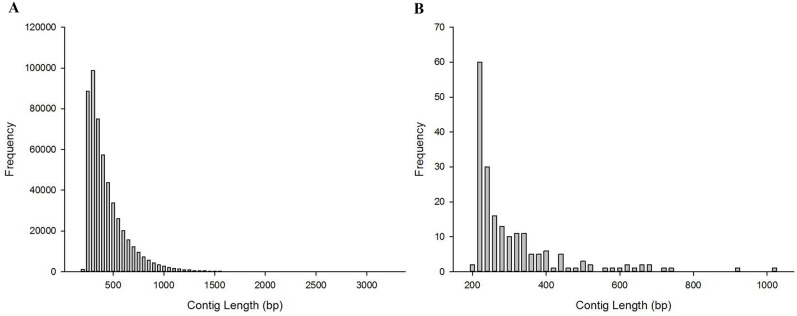
The length distribution of contigs **(A)** The length distribution of the assembled ^*^K-mers-60. **(B)** The length distribution of candidate Y chromosome-specific fragments.

The clean data from M1 library totaled 54.17 Gb, including 361,160,556 PE reads, and the clean data from F’-Mix library totaled 5.91 Gb and contained 38,487,986 PE reads (Figure 1). In order to obtain candidate Y chromosome-specific fragments, three steps were performed. Firstly, male K-mers-60 were generated. After cutting the clean reads of M1 library, 361,160,556 K-mers-60 were obtained. Then male K-mers-60 were aligned to the female reference genome. There were two types of alignment results. The first one was that some male K-mers-60 matched to the female reference genome, which meant that they existed in the both sexes, and the number of K-mers-60 in this type was 89,460,228, and we discarded those K-mers-60. The second one was that some male K-mers-60 could not map to the female reference genome, which meant that they were specific for M1 individual, we kept those K-mers-60 for future analysis and named them as ^*^K-mers-60, the number of ^*^K-mers-60 was 271,700,328. Secondly, we used Iterative De Bruijn Graph Assembler (IDBA) to assemble ^*^K-mers-60, and obtained 515,091 male-specific contigs. Those contigs were mainly 250-500 bp in length, accounting for 77.10% of the total; contigs with length more than 1500 bp only accounted for 0.26% (Figure 2A). Lastly, the assembled ^*^K-mers-60 were aligned to the F’-Mix library using Bowtie2. The alignment results showed that most contigs of the assembled ^*^K-mers-60 mapped to the F’-Mix library, only 196 contigs did not match to any read of F’-Mix library. Therefore, we hypothesized that those 196 contigs might contain sex-specific fragments on the Y chromosome. The 196 contigs were mainly 220-340 bp in length, accounting for 77.04% in total. Contigs with length more than 580 bp only accounted for 6.63% in total (Figure 2B).

### Validation of sex-specific molecular markers

Specific primers were designed for the 196 contigs and three round primers screening were conducted. After the first round of primers screening, 14 pairs of primers generated a positive PCR product for M-Mix DNA, but not F-Mix DNA. These contigs were Contig-67752, Contig-202477, Contig-218980, Contig-359642, Contig- 364727, Contig-418354, Contig-479363, Contig-489930, Contig-502878, Contig-503524, Contig-505934, Contig- 508242, Contig-511514 and Contig-511915. However, 142 pairs of primers produced PCR products for both M-Mix and F-Mix DNA. Meanwhile, one pair of primers amplified multiple bands in F-Mix DNA but only one band in M-Mix DNA, its corresponding contig was Contig-275834. The rest of the 40 pairs of primers failed to amplify any PCR products in either M-Mix or F-Mix DNA.

Based on the results of the first round of primers screening, 15 pairs of primers (including 14 pairs of primers that generated PCR product only in M-Mix DNA, and one pair of primers that produced different bands in F-Mix and M-Mix DNA) were selected for further validation. After the second round of primers screening, we found that only two pairs of primers produced the same size band in all 12 male individuals, while no objective band was detected in any of the 12 female individuals. These two loci were located on Contig-359642 and Contig-418354. One pair of primers amplified one band in all 12 male individuals, and multiple bands in all 12 female individuals, this locus was located on Contig-275834. The rest of 12 pairs of primers did not have sex-specificity and could not distinguish the sexes of snakehead.

**Figure 3:**
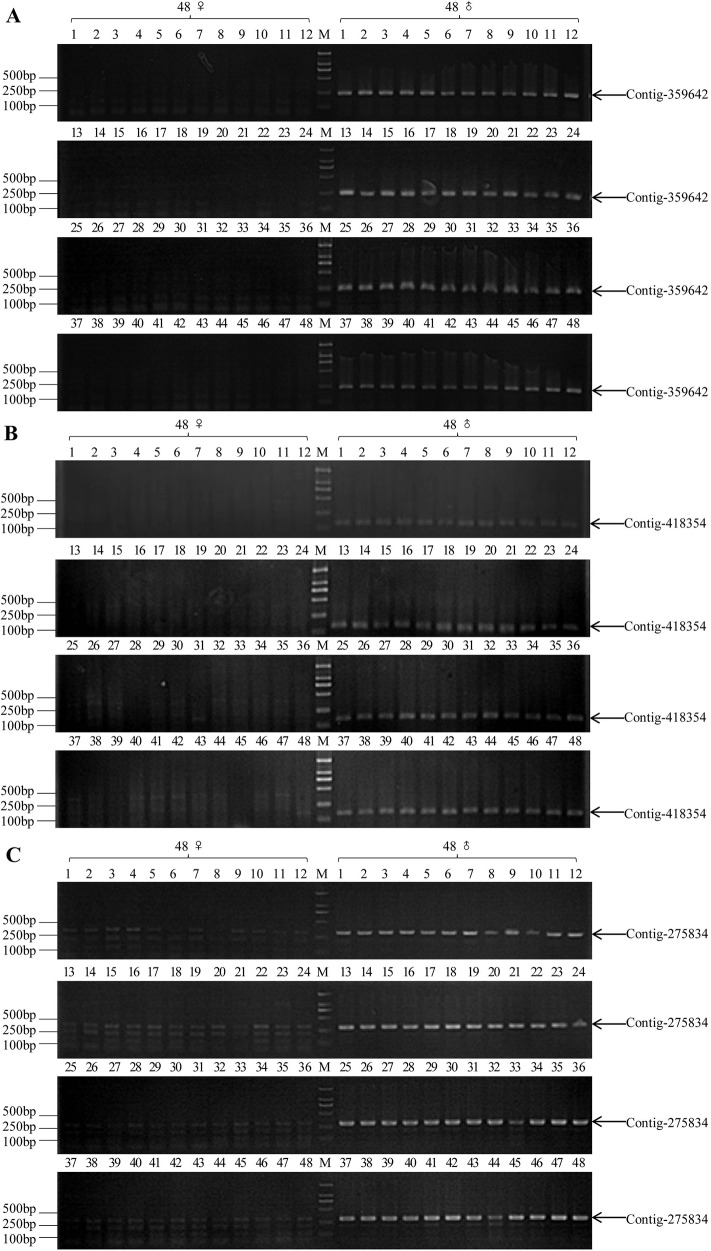
PCR validation results of three pairs of sex-specific molecular markers in commonly wild-caught 48 XY males and 48 XX females (M1-M48 and F1-F48, respectively) **(A)** A 237 bp male-specific fragment (indicated by arrow) was amplified in all male individuals by 359642-F/R primer pair. **(B)** A 158 bp male-specific fragment (indicated by arrow) was amplified in all male individuals by 418354-F/R primer pair. **(C)** A 303 bp male-specific fragment (indicated by arrow) was amplified in all male individuals and multiple bands in all female individuals by 275834X/Y-F primer pair, M: DL2000 DNA marker.

Lastly, we used the screened three pairs of primers to distinguish the genetic sex of 96 male individuals and 96 female individuals. Primers of Contig-359642 (359642-F/R) produced a 237 bp band in all 96 male individuals, while no objective band was detected in any of the 96 female individuals (Figure 3A, Supplementary Figure 1A); and primers of Contig-418354 (418354-F/R) produced a 158 bp band in all 96 male individuals, while no objective band was detected in any of the 96 female individuals (Figure 3B, Supplementary Figure 1B); primers of Contig-275834 (275834-F/R) amplified a 303 bp band in all 96 male individuals, and multiple bands in all 96 female individuals (Figure 3C, Supplementary Figure 1C). In conclusion, these three contigs (Contig-275834, Contig-359642 and Contig-418354) were Y chromosome-specific fragments in snakehead.

### Homologous cloning on X chromosome

Primers of Contig-275834 (275834-F/R) could both obtain objective bands in male and female individuals, while there were also other bands in female individuals. We speculated that the sex differentiation on Contig-275834 was very low, the sequences of primers (275834-F/R) were not specific that might be highly conserved in both sexes. We probably acquire the homologous fragment on X chromosome by homologous cloning.

**Figure 4:**
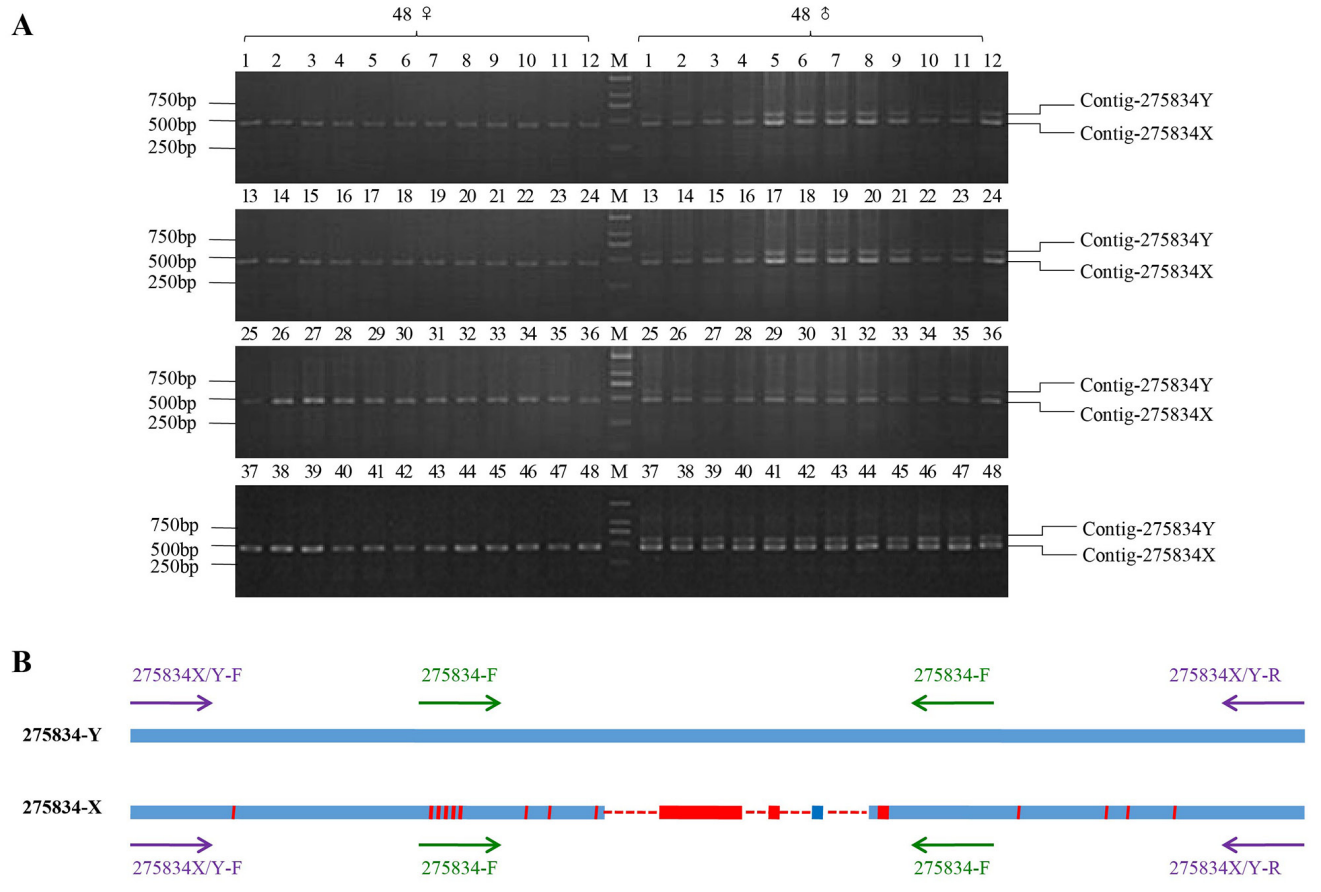
PCR amplification results of homologous cloning on X chromosome **(A)** The PCR amplification results of 275834X/Y-F and 275834X/Y-R in 48 males and 48 females (M1-M48 and F1-F48, respectively). A 458 bp band was expected from PCR amplification in both sexes. Male individuals were also expected to have a 570 bp band that was Y chromosome-specific. **(B)** Sequence alignments of Y chromosome-specific fragment with its homologous fragment on X chromosome. 275834-Y represented the sex-specific fragment on Y chromosome. 275834-X represented the fragment on X chromosome. The red area in the blue line represented the bases which did not map. Red solid line represented the unique regions of 275834-X, and red dotted line represented the deletions of the homologous fragment on X chromosome. The purple arrows were on behalf of the loci of pair primer: 275834X/Y-F and 275834X/Y-R, and the green arrows were on behalf of the loci of pair primer: 275834-F and 275834-R.

After bioinformatics analysis, we obtained comp-letely conserved flanking sequences in the upstream and downstream of Contig-275834, which both existed in the female and male individual. PCR amplification was conducted on DNA samples from 96 males and 96 females. After 2% gel electrophoresis, a 458 bp band was amplified from all female specimens, while double bands (458 bp and 570 bp) were obtained from all male specimens (Figure 4A, Supplementary Figure 2). Sanger sequencing of positive clones showed that sequences of the 458 bp fragments acquired from the female and male individuals were the same, which located on the X chromosome, and we named them as 275834-X. At same time, we named the 570 bp fragment that only amplified from the male individuals as 275834-Y, which located on the Y chromosome. The alignment results of 275834-X and 275834-Y showed that there were 112 bp In/Dels between them. Comparing with 275834-Y, there were four deletions (16 bp, 7 bp, 68 bp, and 21 bp) on 275834-X, even some short specific sequences existed on 275834-X. In addition to these deletions and short specific sequences, there were many SNPs between 275834-X and 275834-Y. In general, 275834-X with 458 bp that existed on the X chromosome was highly homologous to 275834-Y with 570 bp that existed on the Y chromosome, showing a similarity of 83.3% (Figure 4B).

## DISCUSSION

This paper reported a NGS-based approach to identify sex-specific markers in snakehead followed by validation of sex-specificity of snakeheads using PCR amplification. Three Y chromosome-specific fragments (Contig-275834, Contig-359642, and Contig-418354) were found and specific primers were obtained to distinguish the sex of snakehead. Additionally, a pair of primers of Contig-275834 (275834X/Y-F and 275834X/Y-R) was exploited to distinguish XX females, XY males, and YY super-males, whose amplification products of different lengths were produced for different sexes. In theory, if the individual is XX female, it will only gain a 458 bp band; if the individual is XY male, it will gain two bands (458 bp and 570 bp); if the individual is YY super-male, it will only gain a 570 bp band.

**Figure 5:**
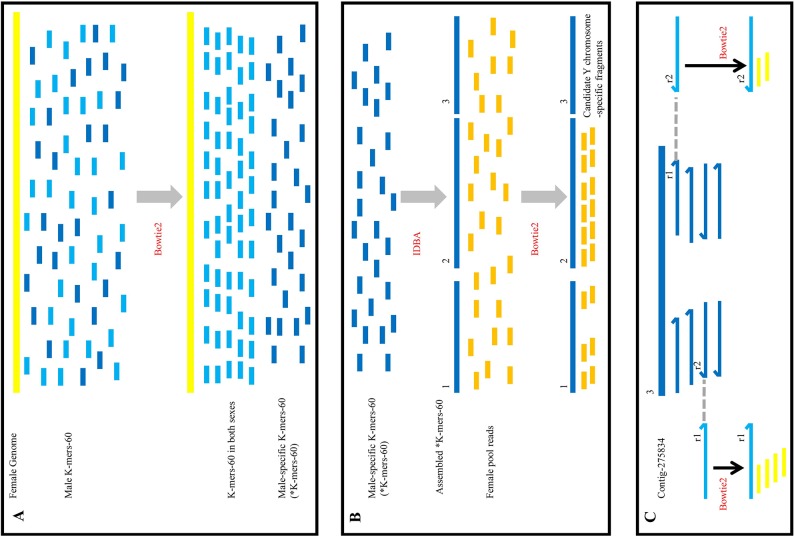
Outline of the screening the sex-specific molecular markers workflow **(A)** The process of subtractive hybridization. Yellow line represented the female reference genome assembled by sequencing reads of F1 library, the short blue (light and dark) lines represented the male K-mers-60. After alignment, light blue lines orderly arranged to the yellow line, it meant that these male K-mers-60 existed in both sexes and would be discarded; dark blue lines randomly distributed, it meant that these male K-mers-60 were male-specific (^*^K-mers-60), which were remained in our further analysis. **(B)** The process of the enrichment of target fragments. ^*^K-mers-60 were assembled (the long dark blue lines), and aligned to reads from the female pool (the short dark yellow lines). There were three types of result. The first type (“1”) was that a little of reads aligned to the assembled ^*^K-mers-60; the second type (“2”) was that a mass of reads aligned to the assembled ^*^K-mers-60; the last type (“3”) was that no read aligned to the assembled ^*^K-mers-60. We discarded the first and second types of fragments, and remained the third type of fragments. Then we verified them by PCR amplification. **(C)** The process of homologous cloning on X chromosome. Contig-275834 (the long dark blue line) was Y chromosome-specific fragment, which was the third type. Contig-275834 aligned to reads from the male library. According to the in pair property of sequencing reads, we obtained the flanking sequences at both ends of Contig-275834 (r1 in the left and r2 in the right, the light blue line represented). Then we utilized them as new references. Female K-mers-60 from the female library (the yellow short line) aligned to the reference respectively, complete alignments were adopted. Alignment results showed there were highly homologous regions between the X and Y chromosomes.

The rationale of this method is that significant differences in sex chromosomes are readily discovered by aligning the male and female genomes. This method is simple and efficient, and it involves three basic steps (Figure 5). The first step is subtractive hybridization. The male reads were spitted into 60 bp (K-mers-60) and aligned to the female reference genome coming from female reads, unaligned male K-mers-60 (^*^K-mers-60) were kept in next filter process. Then ^*^K-mers-60 were assembled to generate male-specific contigs. In theory, these contigs not only contained specific regions on the Y chromosome, but also autosomal loci that differed between males and females. In addition, random In/Dels in sequencing reads of the male and female individual also produced false positive contigs. The second step involves the enrichment of target sequences. Male-specific contigs deriving from the first step were aligned to the sequences from the female pool library. Contigs that could map to any read from the female pool library were discarded, and the remaining contigs were those that most likely derived from the Y chromosome. Through subsequent PCR validation, the sex-specific loci on the Y chromosome were obtained. The last step involved counter-parting. Target Y chromosome-specific contig was aligned to the clean reads from the male library, according to the in pair property of sequencing reads, we obtained the flanking sequences in both ends of the target contig. Then the female K-mers-60 were aligned to the flanking sequences to find the conserved sequences existing in both sexes. Finally, specific primers were designed according to those conserved sequences, showing that homologous fragment on X chromosome could be obtained through PCR amplification (Figure 4A), and there were high similarity between the fragment on Y chromosome and its homologous fragment on X chromosome (Figure 4B).

Comparing to SSRs, AFLP or RAD sequencing, there are three advantages of our NGS-based method. One advantage is that the sequencing data generated by NGS allows for the rapid creation of PCR primers and subsequent validation of sex-specific markers. A large number of SSR markers previously need to be isolated and screened before they are used to identify sex-specificity, which is a painstaking method that takes time. When using AFLP technology to identify sex-specific markers, there is a high demand on the purity and quality of DNA. At the same time, its detection range in the genome is only 100-500 bp for the limitation in the selective nucleotides. Therefore, the regions of sex differences in fish genome may escape from AFLP scanning. In fact, it is unable to detect the presumed sex-associated AFLP fragments in many fishes [32, 33, 34]. The second advantage is that our method does not depend on restriction enzyme sites. When using RAD-sequencing to identify sex-specific markers, sometimes one restriction enzyme may fail, and it needs to switch to another enzyme that cuts more frequently in the genome. It greatly increases the difficulty and cost of finding sex-specific markers [35]. The third advantage is that the sex-specific loci uncovered by our method are significant differences between the sexes, not just single-base difference. It not only harbors abundant SNPs, but also displays a wide range of In/Dels between male-specific fragment on Y chromosome and its homologous fragment on X chromosome (Figure 4B). We easily validated X and Y loci through genotyping with a single PCR primer pair. Once the fin can be obtained from one fish in juvenile, simple PCR amplification can efficiently distinguish XX females, XY males, and YY super-males of an artificial breeding population according to different bands in different sexes.

In our study, we found that most of primers (141 pairs) produced amplification bands with similar size in the male and female pools. This situation might be associated with the location of the PCR primers. In fact, amplification bands with same size in both sexes do not necessarily mean that there are no sequence differences between them, it just means that these differences cannot be detected by this simple genotyping method. Even if there are significant differences in regions on X and Y chromosomes, this could not rule out that, in some local regions, such as primer loci, the DNA sequences on X and Y chromosomes are highly similar. The lower differentiation of sex chromosomes are, the more possible this situation will be.

In addition, we found that some primers (40 pairs) did not yield any bands in either males or females. This might relate to the generation of the male-specific contigs, which was mentioned in Acquisition of the candidate Y chromosome-specific fragments. There were some splicing errors using IDBA to assemble these 60 bp short fragments, and primers based on these “coined” contigs would fail to amplify any products. In support of this, subsequent analysis uncovered that about 125 splicing errors existed in 25,180 contigs (data not shown).

The method described in this article can be further optimized. Firstly, methods to obtain different genomic fragments from the male and female can be more efficient and convenient. We assembled female sequencing reads and obtained 507,119 scaffolds, which came to 646 Mb. Our results was consistent with the results that Xu et al. reported, which a draft *C. argus* genome of 615.3 Mb was assembled, with a contig N50 size of 81.4 kb and a scaffold N50 size of 4.5 Mb [31]. However, the assembly quality of our genome was relatively poor since the Contig N50 and Scaffold N50 are much low, and large amounts of genomic information were lost when using data from a single sequencing library for assembly. In fact, we may make use of the genome that Xu et al. reported, or directly utilize the clean data (150 bp) from the female library and build a series of female reference genomes, then K-mers-60 from the male library are aligned to these series of female reference genomes, gradually eliminating K-mers-60 which are existing in both sexes. As a result, the coverage of the female genome can greatly improve the efficiency to detect male-specific loci, and reducing large numbers of false positive contigs. Additionally, the efficiency for the enrichment of Y chromosome-specific fragments can be further improved. In theory, in a XX/XY sex determination system, female offspring should include all autosomes and X chromosomes that come from their parents, and lack the Y chromosome from the male parent. When using a full-sibling female population to instead of wild females whose genetic relationships are unknown, it is efficient to reduce the false positive fragments, which are the results of differences in autosomes of wild females. Therefore, methods mentioned above will significantly improve the efficiency to enrich the analysis for Y chromosome regions.

We adopted a NGS-based approach to identify sex-specific markers in snakehead, and three Y chromosome-specific fragments (Contig-275834, Contig-359642, and Contig-418354) were identified, a pair of primers of Contig-275834 (275834X/Y-F and 275834X/Y-R) was exploited to distinguish XX females, XY males, and YY super-males according to the rules that different bands were produced for different sexes. These results verified male heterogamety in snakehead, providing a molecular ‘beachhead’ for further exploration of the Y chromosome in this species and underscoring the utility of NGS as a means to rapidly identify sex chromosome systems in non-model species. Meanwhile, identification of sex-specific molecular markers for YY super-males is of great practical significance for all-male breeding strategies in snakehead, and it have tremendous potential for improving the efficiency of breeding practices in other economically critical fish farming industries.

## MATERIALS AND METHODS

### Sample collection and preparation

192 samples (96 male individuals and 96 female individuals, named M1-M96, F1-F96, respectively) came from the Pearl River Fisheries Research Institute, Chinese Academy of Fishery Sciences. Wild-caught snakeheads were collected from different fisheries in RuanJiang County (HuNan Province, China) and XiaoGan City (HuBei Province, China). They are sexually mature individuals, fin tissue was obtained and fixed in 95% ethanol during breeding time. All animal experiments were carried out in accordance with The U.S. Public Health Service Policy on Humane Care and Use of Laboratory Animals.

DNA was extracted using the Universal Genomic DNA Kit (CWBio, China) according to the manufacturer's instructions. The DNAs of M1 and F1 were used for NGS, their data were used for genome assembly and alignments. The same volume of DNAs from F2-F49 were mixed and constituted a female pool, named F’-Mix, the DNA of F’-Mix was also used for NGS, this data was used for discarding the false positive fragments that is individual differences between the male and the female. The same volume of DNAs from M1-M96 were mixed and constituted a male pool, named M-Mix; the same volume of DNAs from F1-F96 were mixed and constituted another female pool, named F-Mix, these two Bulked Segregant Analysis (BSA) pools were used for primer screening. The DNAs of M1-M96 and F1-F96 were used for PCR validation.

### DNA sequencing

The DNAs of M1, F1 and F’-Mix, three samples were built into libraries and used for sequencing. Each DNA sample was separately sonicated using a Fisher Scientific Model 500 Ultrasonic Dismembrator. DNAs were manually size-selected into 300-500 bp fragments using gel electrophoresis, and libraries were prepared according to the methodology originally described in the NEBNext Ultra DNA Library Prep Kit from Illumina. Three libraries were quantified by realtime fluorescence quantitative PCR, and run in three lanes on Illumina HiSeq 2000 using 150 base PE reads (v3 chemistry kit, Illumina, USA). Illumina sequencing and library preparation was performed by the Beijing Genomics Institute.

### Data filter

The raw sequences of three libraries were filtered by AdapterRemoval (https://github.com/slindgreen/AdapterRemoval) [36] to remove contamination and low-quality reads (quality score under 30), and those that passed the quality filter were trimmed to remove the adaptor sequences. The clean sequences were deposited at the NCBI BioProject database, under the accession no. PRJNA357191.

### Assembly of the female genome

We employed SOAPdenovo2 (https://github.com/aquaskyline/SOAPdenovo2) [37] with optimized parameters (pregraph –p 50 −d 2 −K 63; contig –p 20 -m 69 −M 1; scaff −p 50) to link clean reads of F1 library to contigs and scaffolds and build the female reference genome.

### Generation of the male K-mers-60

In the fixed locus of each clean read of M1 library, a 60 bp sequence (from 11 bp to 70 bp) was cut out and generated the male K-mers-60, the forward and reverse reads were treated as independent loci. We chose 60 bp sequence for cutting out on the basis of our previous work (data not shown), and 60 bp had the most likely possibility to remain the candidate Y chromosome-specific fragments when alignments conducted by Bowtie2.

### Acquisition of the candidate Y chromosome-specific fragments

In order to obtain the Y chromosome-specific sequences, we performed three steps. Firstly, contigs from the female genome were used as reference, and sequences of male K-mers-60 were used as query, Bowtie2 (http://bowtie-bio.sourceforge.net/bowtie2/index.shtml) [38] was used for the alignments between the query and reference, and parameters were set to the default values. We collected those K-mers-60 that could not align to the female reference genome, and named those K-mers-60 as ^*^K-mers-60 (^*^ represented unaligned). Secondly, IDBA (http://www.cs.hku.hk/~alse/idba/) [39] was used to assemble these ^*^K-mers-60 data into male-specific contigs with default parameters. Lastly, clean reads (150 bp) of F’-Mix library were used as new reference, the assembled ^*^K-mers-60 were used as new query. Then alignments were conducted between the new query and reference using Bowtie2 with default parameters. Once contig coming from the assembled ^*^K-mers-60 could align to any read from F’-Mix library, this contig would be excluded. The rest of contigs were candidate Y chromosome-specific fragments, which were supposed to be male-specific fragments on Y chromosome.

### Confirmation with PCR amplification

For the candidate Y chromosome-specific fragments, specific primers (Supplementary Table 1) were designed with Primer5 and synthesized by a commercial company (Sangon Biotech, China).

Primers screening included three steps. Firstly, two BSA pools (M-Mix and F-Mix) were used as templates and all primers in Supplementary Table 1 were used for PCR amplification. Each PCR mixture contained 10 μl 2×Taq MasterMix (CWBio, China), 0.8 μl each specific primer (10 μM), and 50 ng DNA, the total volume was 20 μl. The PCR cycling conditions were: one cycle at 94°C for 2 min; 39 cycles at 94°C for 30 s, 51°C for 30 s, and 72°C for 30 s; one cycle of 72°C for 2 min. After separation by 2% gel electrophoresis, primers that only generated PCR product in M-Mix DNA or produced different bands in M-Mix and F-Mix DNA were picked out for further validation.

Secondly, DNAs from 12 male individuals and 12 female individuals and primers picked out from the first screening were used for the second validation. The reaction systems and conditions of PCR amplification were the same as that mentioned above. Similarly, primers that only generated PCR product in all 12 male individuals or produced different bands in all 12 male individuals versus all 12 female individuals were picked out for further validation.

Lastly, we used the screened primers to distinguish the sex of snakehead. DNAs from 96 male individuals and 96 female individuals were used as PCR templates. The reaction system and condition of PCR was the same as mentioned above.

### Homologous cloning on X chromosome

In order to gain the homologous fragment on X chromosome, we adopted a series of bioinformatics analysis. We took Contig-275834 as an example, which was a Y chromosome-specific fragment from the former filter and PCR validation. Firstly, Contig-275834 was used as reference, clean reads (150 bp) of M1 library were aligned to the reference using Bowtie2 with complete alignment strategy. According to the in pair property of sequencing reads, we hoped to gain PE data that one read could completely align to Contig-275834, while its coupled read could not align to Contig-275834. For example, we found in pair reads in M1 library that r2 read completely aligned to the left end of Contig-275834, while its corresponding r1 read could not align to Contig-275834. Thus we obtained 150 bp flanking sequence in the upstream of Contig-275834 in M1 library. Similarly, we obtained 150 bp flanking sequence in the downstream of Contig-275834 in M1 library. These two flanking sequences were respectively used as new reference for further analysis. Secondly, clean reads from F1 library were cut to generate the female K-mers-60. The process was the same as that mentioned in Generation of the male K-mers-60. The female K-mers-60 was used as query and aligned to the upstream and downstream reference from the first step respectively, complete alignments were adopted. As a result, we obtained conserved sequences existing on the Y and X chromosomes that were in the upstream and downstream of Contig-275834.

Specific primers (275834X/Y-F and 275834X/Y-R) were designed according to the perfect matching sequences (Table 2). DNA samples from 96 males and 96 females were used as PCR templates. The reaction system and condition of PCR amplification was the same as that mentioned in Confirmation with PCR amplification. The products of one male and one female were purified by Gel Extraction Kit (Omega, USA), and the purification products were ligated into pMD18-T vectors (Takara, Japan). The ligation products were then transformed into competent *Escherichia coli* DH5α cells (TransGen, China) and cultured at 37°C. Positive colonies were selected and sequenced by a company (TsingKe, China).

**Table 2:** Primers used for homologous cloning

Primer name	Primer sequences (5′-3′)
275834X/Y-F	XXXXXXXXXXXXXXXXXXX^a^
275834X/Y-R	XXXXXXXXXXXXXXXXXXXX^a^

^a^ Note: Because of the economic value of the sex-specific molecular markers in the development of all-male snakeheads breeding industries, so the sequences of the primers used in this report are not public. Please accept our apologies.

## SUPPLEMENTARY MATERIALS FIGURES AND TABLE




