# Supplementary material for: An NGS-based approach for the identification of sex-specific markers in snakehead (Channa argus)
Source: Oncotarget. 2017 Oct 19;8(58):98733–44. doi: 10.18632/oncotarget.21924 (PMC5716763; doi:10.18632/oncotarget.21924)
Supplement: Supplementary file 1 [file oncotarget-08-98733-s001.pdf]

An NGS-based approach for the identification of sex-specific markers in snakehead (*Channa argus*)

SUPPLEMENTARY MATERIALS

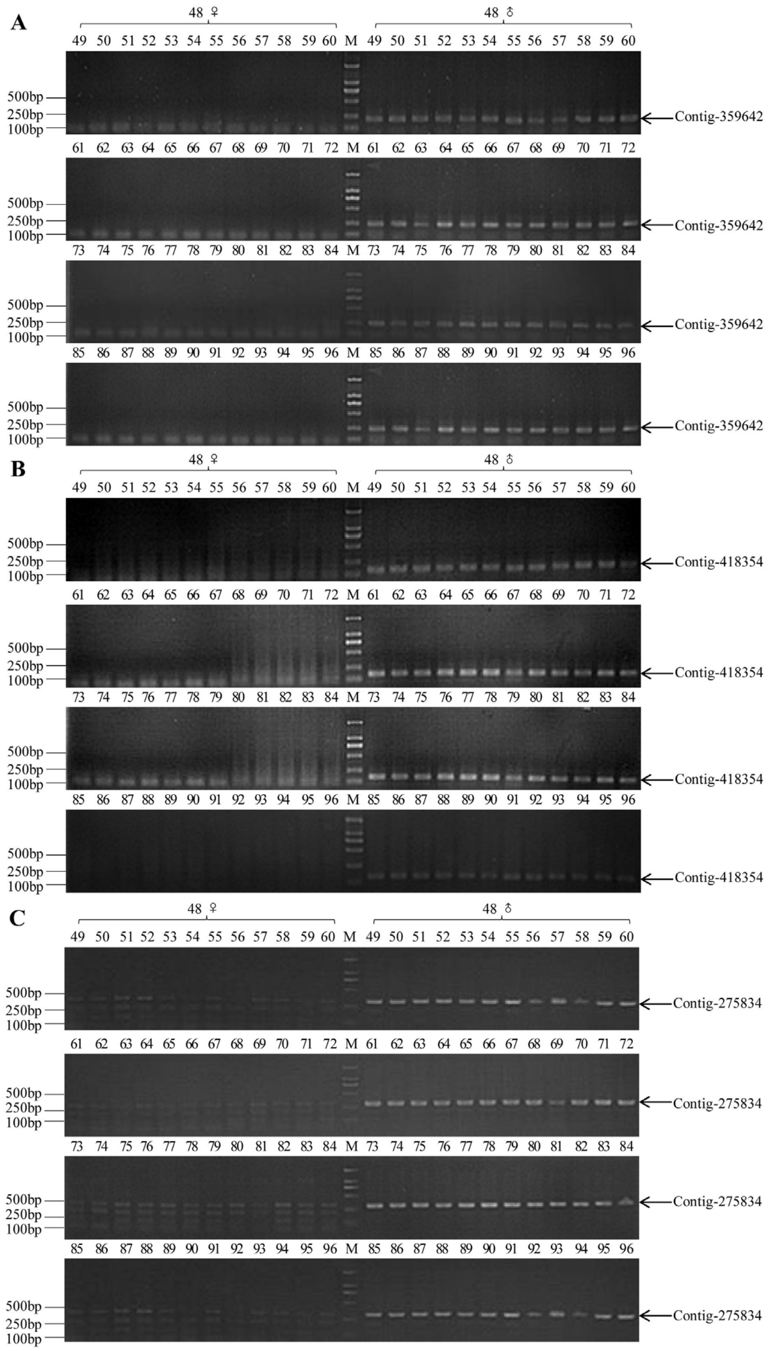

Supplementary Figure 1: PCR validation results of three pairs of sex-specific molecular markers in commonly wild-caught 48 XY males and 48 XX females (M49-M96 and F49-F96, respectively). **(A)** A 237 bp male-specific fragment (indicated by arrow) was amplified in all male individuals by 359642-F/R primer pair. **(B)** A 158 bp male-specific fragment (indicated by arrow) was amplified in all male individuals by 418354-F/R primer pair. **(C)** A 303 bp male-specific fragment (indicated by arrow) was amplified in all male individuals and multiple bands in all female individuals by 275834-F/R primer pair, M: DL2000 DNA marker.

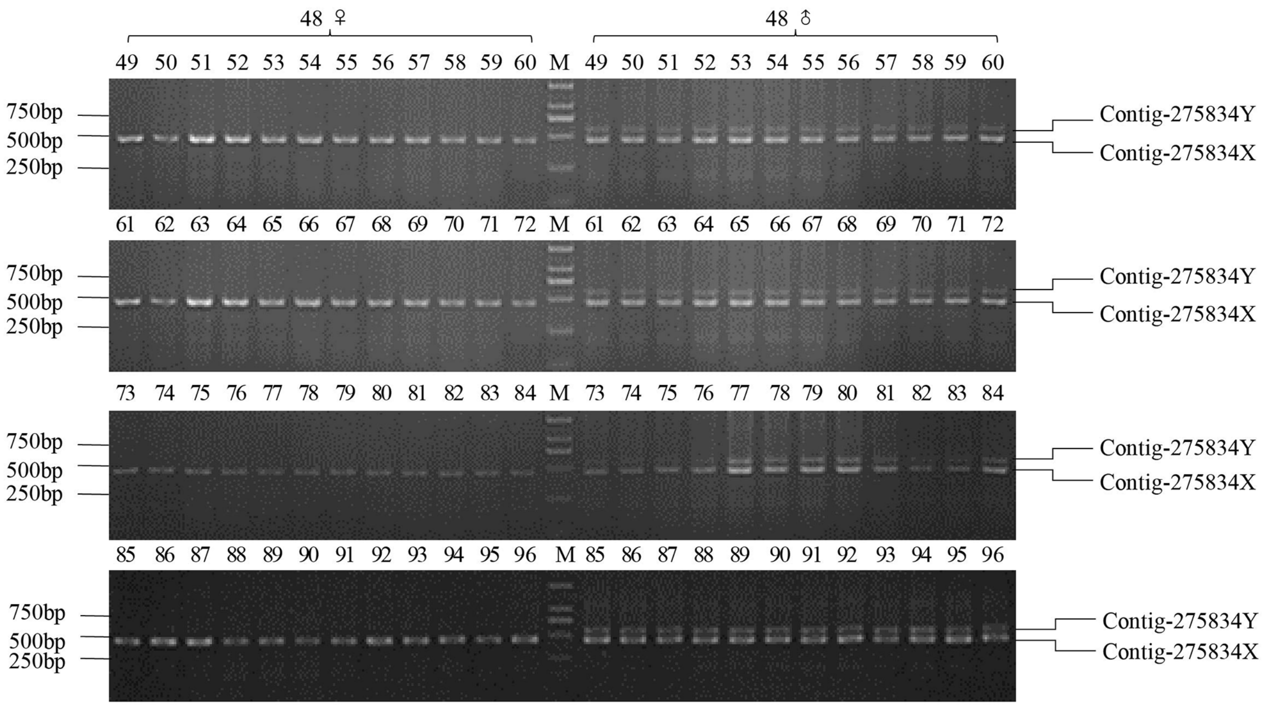

Supplementary Figure 2: The PCR amplification results of 275834X/Y-F and 275834X/Y-R in 48 males and 48 females (M49-M96 and F49-F96, respectively). A 458 bp band was expected from PCR amplification in both sexes, male individuals were also expected to have a 570 bp band that was Y chromosome-specific.

Supplementary Table 1: Primers used for PCR validation.

See Supplementary File 1
