# Supplementary material for: An NGS-based approach for the identification of sex-specific markers in snakehead (Channa argus)
Source: Oncotarget. 2017 Oct 19;8(58):98733–44. doi: 10.18632/oncotarget.21924 (PMC5716763; doi:10.18632/oncotarget.21924)
Supplement: Supplementary file 2 [file oncotarget-08-98733-s002.doc]

| Contig name | Primer name | Sequences(5’-3’) | Size (bp) | F-Mix | M-Mix |
| --- | --- | --- | --- | --- | --- |
| Contig-107794 | 107794-F | CCATTGCTTCAAAACTGTGC | 286 | √^a^ | √ |
|  | 107794-R | GCAAAAGTTCACCAGTCACAAG |  |  |  |
| Contig-114881 | 114881F | ATAAACACAAACCCAAAGAAT | 473 | √ | √ |
|  | 114881R | ACATAGTCACAAGCACCATAAT |  |  |  |
| Contig-119571 | 119571F | ATTGGGAAAGAGGTTGCTG | 372 | √ | √ |
|  | 119571R | ATTGCCAGCCTTGATGATAA |  |  |  |
| Contig-122680 | 122680F | GTCATAGACAGTTGGGGAGAG | 289 | √ | √ |
|  | 122680R | TGTATTTTTAGTCGTGATGGG |  |  |  |
| Contig-128010 | 128010F | GACAAAAAAACTCTCTGGAAA | 439 | √ | √ |
|  | 128010R | CACAACCCTAAACAGGATAAG |  |  |  |
| Contig-141901 | 141901F | AGATTTGACCTGCTTTTTG | 349 | √ | √ |
|  | 141901R | AAACTTTCCTTTGTATCACTG |  |  |  |
| Contig-145136 | 145136F | GGTTTCCTCCAGTTTCTTTG | 286 | √ | √ |
|  | 145136R | ATTTCCCATCATAGCCAGC |  |  |  |
| Contig-15226 | 15226F | GTTCAAACACCATCTTCTTAGTC | 797 | √ | √ |
|  | 15226R | ATTTAGTGTGGCTGATTTGG |  |  |  |
| Contig-162466 | 162466F | ATCCTCCTCTCAGCCAGT | 376 | √ | √ |
|  | 162466R | ATCTACCATTACGAGACATTACT |  |  |  |
| Contig-164501 | 164501F | CCACCACCGTGCTGCTC | 304 | √ | √ |
|  | 164501R | CTGTCAGTGATGGATGGATTG |  |  |  |
| Contig-164843 | 164843F | CTGTCAGCAGAACACACCAAA | 234 | √ | √ |
|  | 164843R | CACCCCCCGTTTATGGAT |  |  |  |
| Contig-167211 | 167211F | CTCCTCAATAGGTTTGCTGC | 395 | √ | √ |
|  | 167211R | CTCCAGGCTCTTCCTCTCC |  |  |  |
| Contig-168672 | 168672F | CCTACAGCCAGCGGGTTT | 349 | √ | √ |
|  | 168672R | CGCACACATTTTTGCTTCG |  |  |  |
| Contig-177938 | 177938F | GCCCTAAAGACTGAGGTAAGAG | 318 | √ | √ |
|  | 177938R | CTAAGGCACTTGACACCACA |  |  |  |
| Contig-201200 | 201200F | GGGACTTGAGTCACATTTATATC | 263 | √ | √ |
|  | 201200R | GAAGTGGCACAAAAATCG |  |  |  |
| Contig-202477 | 202477F | TATGTAGCCACCACTGTCTGC | 180 | /^b^ | √ |
|  | 202477R | GGGTGGAGTCTGTCCTGCT |  |  |  |
| Contig-202522 | 202522F | GTGGTATGAGACAAATAGTGC | 288 | √ | √ |
|  | 202522R | TACTCACACAAAAAAGCGT |  |  |  |
| Contig-210225 | 210225F | ACCAACCAGTCAAATAGCAT | 310 | √ | √ |
|  | 210225R | CCTGGTTTTGTTCAGTTTGT |  |  |  |
| Contig-210581 | 210581F | CCTACACTCGGGGGAAG | 368 | √ | √ |
|  | 210581R | CCCTTGAACTTGTTTTGTCCA |  |  |  |
| Contig-211010 | 211010F | AATAGATGTTCAGTCTTGTTTGT | 188 | √ | √ |
|  | 211010R | GAAAATCAGGAGAGAGAGGT |  |  |  |
| Contig-218752 | 218752F | TGTTTGACATACACTTACCCATC | 196 | √ | √ |
|  | 218752R | GAACCACCTTTCTTCCCATT |  |  |  |
| Contig-218980 | 218980F | TTTCAAAACAAATACTCCCTC | 155 | / | √ |
|  | 218980R | TGAACCAGGCACAAACTTA |  |  |  |
| Contig-223254 | 223254F | GGTCTTCCTGACAGCCTTC | 340 | √ | √ |
|  | 223254R | CAACTCCTGGTTTGAAGCAC |  |  |  |
| Contig-231848 | 231848F | AAAAAACTTCAGACCAAACC | 222 | √ | √ |
|  | 231848R | TGGACACTATCAAAACAACC |  |  |  |
| Contig-237623 | 237623F | TAGTCACCACTTTCCGTAAT | 210 | √ | √ |
|  | 237623R | ACACATCTTGACCATTTTTG |  |  |  |
| Contig-239980 | 239980F | GTCCCTTCCAATCTAATACAT | 173 | √ | √ |
|  | 239980R | ACATAAAACAAAGAACAGCAG |  |  |  |
| Contig-240203 | 240203F | TTGAATGCCCACAAACCTG | 180 | √ | √ |
|  | 240203R | CAGGAGGGATGACCAACACT |  |  |  |
| Contig-246494 | 246494F | GACTCAGGGCATCGGTTG | 306 | √ | √ |
|  | 246494R | TGGCACTGACAAAAACAACAC |  |  |  |
| Contig-252637 | 252637F | CGTAGTGAAACACATAACCGT | 264 | √ | √ |
|  | 252637R | GGGATGTTTTACAGGCACT |  |  |  |
| Contig-257184 | 257184F | ATTATTAGGAACTTGTGGTAGAA | 275 | √ | √ |
|  | 257184R | TGACAGAGGAGTTCAAGGTG |  |  |  |
| Contig-267808 | 267808F | AAGGTTGGCGTTTTAGTGTA | 310 | √ | √ |
|  | 267808R | TAACATTTTTTTCATTTCACTTC |  |  |  |
| Contig-268162 | 268162F | TAGTTACAACATTTTACCATCTG | 205 | √ | √ |
|  | 268162R | AAGTCCAGCAAAATCAAATA |  |  |  |
| Contig-270339 | 270339F | GTCAGAGAAAGATGGTGGTT | 228 | √ | √ |
|  | 270339R | ACAGAAATGTCAAGTAACAAGTAT |  |  |  |
| Contig-272531 | 272531F | GTCTGCTGTTTGTAGCCATT | 288 | √ | √ |
|  | 272531R | TGGATCTTCACATTAATAGCTG |  |  |  |
| Contig-274699 | 274699F | CTGAGAGGAATAAAAGAGCAAAC | 312 | √ | √ |
|  | 274699R | TCTGGTGATTTGGCTGCTC |  |  |  |
| Contig-275834^c^ | 275834F | XXXXXXXXXXXXXXXXXXX^d^ | 303 | √ | √ |
|  | 275834R | XXXXXXXXXXXXXXXXXXXX |  |  |  |
| Contig-278319 | 278319F | TGTTCCCTATTTCGGCT | 240 | √ | √ |
|  | 278319R | ACAGTGATTTGTACCATTCTTAT |  |  |  |
| Contig-285766 | 285766-F | CTCCCTGTTAGGATGTGCTG | 282 | √ | √ |
|  | 285766-R | ACTGCCACCATCTGATTTATT |  |  |  |
| Contig-287288 | 287288-F | GAAGGACATTTATATTCACTCTCAGC | 238 | √ | √ |
|  | 287288-R | GTGTTTGGATTCATTTTCCCTAA |  |  |  |
| Contig-292283 | 292283-F | CGATGATGTGATTGTGTATGTAGG | 238 | √ | √ |
|  | 292283-R | CTGGAATAATCTTGCCCTGAC |  |  |  |
| Contig-292296 | 292296-F | GAGGACATTAACCCGTAAACACC | 241 | √ | √ |
|  | 292296-R | CTTCTCTCACCCCATCACATTC |  |  |  |
| Contig-297651 | 297651-F | TCCAACTACTTTTACAACCCA | 193 | √ | √ |
|  | 297651-R | TAAACATCCTGTCAGCATAGC |  |  |  |
| Contig-300986 | 300986-F | CACCACTGGGATTCGTC | 230 | √ | √ |
|  | 300986-R | TAACTACTCTTACCCTTATCCAG |  |  |  |
| Contig-305448 | 305448-F | TAGATGTATTACTATTGCTGAGGG | 172 | / | / |
|  | 305448-R | GTATTGCAAAGAAGTGTAAAAAAC |  |  |  |
| Contig-309550 | 309550-F | AATCTAAAGTTTAATTGGCTGC | 221 | √ | √ |
|  | 309550-R | TGCAACATGATGTTTATGTAATG |  |  |  |
| Contig-315477 | 315477-F | TACGCTAACAGTTTACAGACC | 175 | √ | √ |
|  | 315477-R | AGCCGTGACAATGTGAGA |  |  |  |
| Contig-317944 | 317944-F | CCAGTGTCCTCTGAGCATT | 265 | √ | √ |
|  | 317944-R | TGAAAATGTGGCACTGAGAG |  |  |  |
| Contig-320983 | 320983-F | AAAGTGTTAGACCAGAAAATGTTG | 248 | / | / |
|  | 320983-R | GGATGGAGTAGTGTTTTTGGTT |  |  |  |
| Contig-322865 | 322865-F | CACACATTCCCAAGGTAAAA | 189 | √ | √ |
|  | 322865-R | CTCAAAGATGACTATGAAACACA |  |  |  |
| Contig-324326 | 324326-F | GGTCTGTTGGTGTAACTCTGTC | 229 | / | / |
|  | 324326-R | AGTGTCCTGACCTCTAACCC |  |  |  |
| Contig-324724 | 324724-F | CAGTTTTTATCAGGTCAGCAG | 283 | √ | √ |
|  | 324724-R | CCTGTCTCTGTTTTAGGTTCA |  |  |  |
| Contig-325512 | 325512-F | AGTAAAAGTTCAAAAGTAAGAT | 228 | √ | √ |
|  | 325512-R | CATTGTAACTAACAGGCTC |  |  |  |
| Contig-326817 | 326817-F | CATCAGATGTAGTCTAAGAGTCAG | 226 | √ | √ |
|  | 326817-R | GAAGGAGAAGCCATATGC |  |  |  |
| Contig-327064 | 327064-F | TGGTGCTGTTGTTGGAGT | 285 | √ | √ |
|  | 327064-R | GTATCACTGGTAGAACCTATTTG |  |  |  |
| Contig-330563 | 330563-F | GTGTTATGTCTCACTCGCTTTC | 258 | √ | √ |
|  | 330563-R | TTGACACCGTTGATACTGAATA |  |  |  |
| Contig-338767 | 338767-F | ATCATTTTAGTTTCCACCACC | 237 | √ | √ |
|  | 338767-R | TTCCACACATTGAACAGCC |  |  |  |
| Contig-36183 | 36183-F | CTCTGTCCAACCAGCATAGT | 278 | √ | √ |
|  | 36183-R | ACACAGACCTCATCAGCAAC |  |  |  |
| Contig-41703 | 41703-F | AGTGAAGTTGAATAACAAAGGC | 384 | √ | √ |
|  | 41703-R | GTCAATAGAGGGAGTGTGTGC |  |  |  |
| Contig-342704 | 342704-F | GTGGGGCCAAAACACAAAC | 205 | √ | √ |
|  | 342704-R | GTGACCAAAGCCCACGAGG |  |  |  |
| Contig-347564 | 347564-F | GTCATCCAGGCAACCC | 189 | / | / |
|  | 347564-R | ATCTATGAGTGAGTAGTTTTTTG |  |  |  |
| Contig-352868 | 352868-F | GCTTTTCCACATTCTTTACACC | 221 | √ | √ |
|  | 352868-R | GTCAGTGGACACACACGCTC |  |  |  |
| Contig-353607 | 353607-F | ATATCCACCAACAATATGAG | 204 | √ | √ |
|  | 353607-R | TGTTTTAGGTTTGTATTATCAG |  |  |  |
| Contig-355540 | 355540-F | CCATTTCTCGCTCTGCCTC | 224 | √ | √ |
|  | 355540-R | TGGTTCATTCTTTGCCTTTTG |  |  |  |
| Contig-359642 | 359642-F | XXXXXXXXXXXXXXXXXXXXX^d^ | 237 | / | √ |
|  | 359642-R | XXXXXXXXXXXXXXXXXXXXX |  |  |  |
| Contig-362956 | 362956-F | ACTGGAACCAGAACAGAACA | 241 | / | / |
|  | 362956-R | ACTACAAAAACATGAAACTCTGC |  |  |  |
| Contig-364727 | 364727-F | GTAGCATCAGTACCATAGAAAAC | 223 | / | √ |
|  | 364727-R | GCACTTTTCTGTGTAGAGGTT |  |  |  |
| Contig-374675 | 374675-F | AAGGACACACACACAAAAAA | 161 | / | / |
|  | 374675-R | GATATAAGACAGGTTGAGAGATG |  |  |  |
| Contig-378682 | 378682-F | AGACACCCATTTGTTTCCTTG | 193 | / | / |
|  | 378682-R | CATTATCATTCTCCCCCCAT |  |  |  |
| Contig-389149 | 389149-F | AACTGATTCTCCTGTCGTATTA | 196 | / | / |
|  | 389149-R | GGATGGGTCTCTGTGATTG |  |  |  |
| Contig-389320 | 389320-F | GACCTTCAGTCTGTAGTTTGTTCTC | 215 | √ | √ |
|  | 389320-R | GGTGCCCTAAAAGGACAAAG |  |  |  |
| Contig-392232 | 392232-F | TCCATGGCAACCCAAGAG | 215 | √ | √ |
|  | 392232-R | TATTTGGAGTGAGATTGTTGAGC |  |  |  |
| Contig-393240 | 393240-F | AGTGCATTTGAATGTCCAGTAC | 205 | √ | √ |
|  | 393240-R | TGTTTTGTGCTGTGTCCGT |  |  |  |
| Contig-395629 | 395629-F | AACCCAATGCTTTACCCAC | 243 | √ | √ |
|  | 395629-R | ACGCAGACCTATGCAGAGAT |  |  |  |
| Contig-398341 | 398341-F | CAAGAGGAAGGCCAGAGGTC | 226 | √ | √ |
|  | 398341-R | AAAACAGGAGAAGAGAATCAACAG |  |  |  |
| Contig-401107 | 401107-F | TTATCAAGGAAAAATACACAGA | 162 | √ | √ |
|  | 401107-R | CAAATGCTACCCAGACAGA |  |  |  |
| Contig-402131 | 402131-F | ATGATGAAATATTTGAACCA | 158 | / | / |
|  | 402131-R | ATACTTGACCTGATGAACTG |  |  |  |
| Contig-403128 | 403128-F | GTTTTGAAAATGATTCCTTAT | 228 | √ | √ |
|  | 403128-R | TTTATTTTCAGTGGGCTCT |  |  |  |
| Contig-404596 | 404596-F | GTCTCTCACACTGACGGGA | 208 | / | / |
|  | 404596-R | CAAACTTTCAAGCAACACAG |  |  |  |
| Contig-404923 | 404923-F | TCCAAATTAAATTCCTTCTT | 166 | / | / |
|  | 404923-R | GTTCAATCACAAAATCACAGT |  |  |  |
| Contig-407211 | 407211-F | GTGTCCAAATCCAACTCATCC | 235 | √ | √ |
|  | 407211-R | GTTGTCAAAGTGGTTTAGTTGTTAT |  |  |  |
| Contig-409353 | 409353-F | TTGAAACTTTTACAACCTT | 190 | √ | √ |
|  | 409353-R | ATGTTCCTAATCTTTTTCTAC |  |  |  |
| Contig-411173 | 411173-F | CAGAGCAGATGAGACACAGC | 194 | √ | √ |
|  | 411173-R | ATCAGATAATTTCAAATTTTTCG |  |  |  |
| Contig-413359 | 413359-F | ATCAGATAATTTCAAATTTTTCG | 253 | √ | √ |
|  | 413359-R | ACAAAAAAACCCCAAAAAAC |  |  |  |
| Contig-415736 | 415736-F | CAGAGTTACACCAACAGACC | 236 | √ | √ |
|  | 415736-R | GAGGAAGACAGACTGAGGAG |  |  |  |
| Contig-416549 | 416549-F | TACCAAAATCAACACAAAAC | 164 | √ | √ |
|  | 416549-R | TTCACCAGACTTCAACCTA |  |  |  |
| Contig-418354 | 418354-F | XXXXXXXXXXXXXXXXXXXX^d^ | 158 | / | √ |
|  | 418354-R | XXXXXXXXXXXXXXXXXX |  |  |  |
| Contig-421780 | 421780-F | CACTCGCAAAGTGTGATGAG | 199 | √ | √ |
|  | 421780-R | GAATAGTGAATGAGGGATGGA |  |  |  |
| Contig-422388 | 422388-F | CAATAACACAAAATCTCATCAC | 205 | √ | √ |
|  | 422388-R | CTTACATTCTTTACTGTAAACCAG |  |  |  |
| Contig-422535 | 422535-F | TGAGTTTTCCAGTTTCGGTG | 242 | √ | √ |
|  | 422535-R | TGCTTCCCCCGAGTGTAG |  |  |  |
| Contig-432799 | 432799-F | TTACCAAATCTCCATCACCC | 206 | √ | √ |
|  | 432799-R | TTCCTGAGATGTTCTGCCTT |  |  |  |
| Contig-438253 | 438253-F | GGCAAAATGTATTCATCACTC | 177 | √ | √ |
|  | 438253-R | TTGACTGTTTATGTTTGCTCC |  |  |  |
| Contig-442782 | 442782-F | GTCTAACAATAAGGGTGAAAGC | 240 | √ | √ |
|  | 442782-R | CTTCAGGCATCAAACTCTAAAT |  |  |  |
| Contig-448197 | 448197-F | GCATAAATAAAGTGAAATACAAAGC | 191 | √ | √ |
|  | 448197-R | CTTCCGTTTATCCAGAGTCG |  |  |  |
| Contig-449069 | 449069-F | CCCTTCAGTGTTTTTCTCTT | 204 | √ | √ |
|  | 449069-R | ACGGCGCAAATGATCTA |  |  |  |
| Contig-453747 | 453747-F | TGTCTTGAACTTCACTTGCC | 222 | / | / |
|  | 453747-R | GTGTTGACAGATAATGGATGG |  |  |  |
| Contig-454704 | 454704-F | GATCTAAATGACAATTAAAAAACTA | 151 | √ | √ |
|  | 454704-R | AGCCAAAGACTATTCCTGTG |  |  |  |
| Contig-454926 | 454926-F | GGAACTTCATACCTGCGA | 199 | / | / |
|  | 454926-R | GTTTGAAAGTATATTGTTTAGGC |  |  |  |
| Contig-455224 | 455224-F | AAGAGAAACACACTATCAGGCTA | 192 | / | / |
|  | 455224-R | TGTATCAGCCGAGTGCTTAC |  |  |  |
| Contig-456739 | 456739-F | GCTAACCAGCTACTCATTCC | 181 | / | / |
|  | 456739-R | TGTGTTGCCTAAATAACCTGT |  |  |  |
| Contig-456826 | 456826-F | TTACAGGAACAAACCAAATG | 232 | √ | √ |
|  | 456826-R | ACTCAACTTTCTTCTGCTGC |  |  |  |
| Contig-458822 | 458822-F | AAGACTGTTCCAAAGAATATAG | 185 | √ | √ |
|  | 458822-R | TTGTTGAGGTTTTATTTTTC |  |  |  |
| Contig-459141 | 459141-F | TAAAAAGGTGGTGATGATAAAC | 153 | / | / |
|  | 459141-R | GCACATAACACAGGTGAAGAT |  |  |  |
| Contig-460922 | 460922-F | GTGACTAATTCACTGATGCAAC | 197 | √ | √ |
|  | 460922-R | TGAAGTATTTTCCACAACACA |  |  |  |
| Contig-48412 | 48412-F | TCTGTCTTCACCCGCTG | 255 | √ | √ |
|  | 48412-R | CACATTCTCAACCTCAACTCTA |  |  |  |
| Contig-48520 | 48520-F | CTGCCTTCATTATGGACA | 300 | √ | √ |
|  | 48520-R | ACTTTTAGTTCATTTAGCCT |  |  |  |
| Contig-461053 | 461053-F | AATATAAATAATCACAAGTATGCAG | 201 | √ | √ |
|  | 461053-R | GTGCACGCTGCTCTATTC |  |  |  |
| Contig-463248 | 463248-F | CGAGGAAACGAAGGATGTC | 215 | √ | √ |
|  | 463248-R | GTGTTAAACCAGCTAAACAGCC |  |  |  |
| Contig-464873 | 464873-F | TTAGTAGGGTAAACTTGTGGTA | 140 | √ | √ |
|  | 464873-R | GTGTTTTGGGCACATTTT |  |  |  |
| Contig-477033 | 477033-F | CCCAAGTGTGATTGTGTGT | 163 | / | / |
|  | 477033-R | GATCACACATTTTAATTTTTCTA |  |  |  |
| Contig-477371 | 477371-F | CAGTCTTTTCCGAGTTTGTTC | 167 | √ | √ |
|  | 477371-R | AACAAATACGCCACACATCTT |  |  |  |
| Contig-477622 | 477622-F | CTCTTCGGATGAACAGTGC | 180 | / | / |
|  | 477622-R | AACCAGATGGAAACAAAAAG |  |  |  |
| Contig-479363 | 479363-F | TTACATTGTATAAGAAGTTAGTCAC | 178 | / | √ |
|  | 479363-R | CTAAAACACCATAGAGAGACAA |  |  |  |
| Contig-479858 | 479858-F | ATAAATGTCTAACACTCTTGGC | 143 | √ | √ |
|  | 479858-R | TGAAACATTTACAGTGGTGC |  |  |  |
| Contig-481635 | 481635-F | TAGCAACACAATCATTACAAC | 189 | / | / |
|  | 481635-R | ATAAACAGTGAACTCAACTCCT |  |  |  |
| Contig-485261 | 485261-F | GTCAACTAAGAACAGGAAAC | 130 | √ | √ |
|  | 485261-R | AGTGTAATATATGAAGACAAAGAG |  |  |  |
| Contig-488008 | 488008-F | GTCTAAGACCAGATATTGTCATTTC | 205 | √ | √ |
|  | 488008-R | GAGTCTTAGCAGCAGCGAG |  |  |  |
| Contig-489930 | 489930-F | TGTGGCAACAGCAGGAT | 154 | / | √ |
|  | 489930-R | GGACTTCTCTTATACCTCGCT |  |  |  |
| Contig-490503 | 490503-F | ATGTTTTGGTATCTTCTATGTAA | 194 | √ | √ |
|  | 490503-R | GCAGTGTAGTATTTAACCAGG |  |  |  |
| Contig-491396 | 491396-F | AACACTATTTTGGCTTTTACAG | 183 | √ | √ |
|  | 491396- R | ACTGGACTGCTCAGGACAC |  |  |  |
| Contig-491571 | 491571-F | CAATCCTCCTTATCTCTGCTCTTAG | 188 | √ | √ |
|  | 491571-R | TGCTGTTCTGTGAGTCTGGGTC |  |  |  |
| Contig-492133 | 492133-F | CTTTGATAGTTGCTTTTGC | 172 | √ | √ |
|  | 492133-R | ATGTTGTGATGCTTCTTATG |  |  |  |
| Contig-492227 | 492227-F | CACCTAGATGGAGATCAGTG | 206 | √ | √ |
|  | 492227-R | AAGGAAAAGAAGAAGATACATT |  |  |  |
| Contig-492769 | 492769-F | TTGCCCAATACTAAGAACTATGA | 208 | √ | √ |
|  | 492769-R | CTTTCGCAAGGCACTGTAT |  |  |  |
| Contig-493158 | 493158-F | GAGCAAAGACCTGCAAATC | 117 | √ | √ |
|  | 493158-R | GATGATAAAATCCCTAAAGCA |  |  |  |
| Contig-493707 | 493707-F | GTAGCGAGTTGTACCAGTGTAC | 220 | / | / |
|  | 493707-R | CAATATGTGCTCTCTGTCTGCT |  |  |  |
| Contig-494544 | 494544-F | AAGGAAAGGTGTCAGAAAAG | 152 | √ | √ |
|  | 494544-R | CATCAGTCACTACAAAGTTTTATTA |  |  |  |
| Contig-494687 | 494687-F | GAAAAAATAATTGCCCACC | 190 | √ | √ |
|  | 494687-R | TTTAGAAGGCGTAATCACAAT |  |  |  |
| Contig-496276 | 496276-F | AATAAAACTTACAATACAAATCCC | 158 | / | / |
|  | 496276-R | ACATATTTAAGTAGCTCATGCTG |  |  |  |
| Contig-496332 | 496332-F | AAATGGAACTAACTATTGCCTCGTA | 143 | √ | √ |
|  | 496332-R | GCAGAGTGACTGAGCAGAGGG |  |  |  |
| Contig-497593 | 497593-F | GTGCTGTGCCTGCTATAAAAAAG | 149 | / | / |
|  | 497593-R | ACATCACTGACACCAGCCACA |  |  |  |
| Contig-497683 | 497683-F | GGAACACTCGGCACCATAGA | 121 | √ | √ |
|  | 497683-R | GCTTGGAATTGCTTGGTGG |  |  |  |
| Contig-498179 | 498179-F | GTTGCACCTACAAGCACATGA | 145 | / | / |
|  | 498179-R | TTTCCCTTTTCAGAAACCTCA |  |  |  |
| Contig-498214 | 498214-F | TGGTGGTATTTGTTGCATTTATCC | 171 | √ | √ |
|  | 498214-R | CCAGTCCTGCCAGCCTTCTT |  |  |  |
| Contig-498488 | 498488-F | TCATCCCTCCAACTTCCTTAT | 130 | √ | √ |
|  | 498488-R | TGACAGATTATTGATGGAGCAC |  |  |  |
| Contig-499038 | 499038-F | TCCCAGCCTGATGCTTTAC | 201 | √ | √ |
|  | 499038-R | CCCTTTAGTTGGTTGATGGA |  |  |  |
| Contig-499047 | 499047-F | CATTACTTCACAGGAGGACAG | 143 | / | / |
|  | 499047-R | CACGGGGCATATTTTCA |  |  |  |
| Contig-499210 | 499210-F | TCTGATGACTTCACTAATACCA | 168 | √ | √ |
|  | 499210-R | AAAGGGAGTTTTTACACTCC |  |  |  |
| Contig-499219 | 499219-F | ACTGGAAATCAATGGTCAGA | 150 | √ | √ |
|  | 499219-R | GGAATTATGATGCAGTGACTAC |  |  |  |
| Contig-499612 | 499612-F | CATTCCACTGCCTTACCAT | 186 | √ | √ |
|  | 499612-R | ACACAAAAACACACCAGAGC |  |  |  |
| Contig-500597 | 500597-F | CAGGCAGGAAGGTAACAAAG | 190 | √ | √ |
|  | 500597-R | TAGCCTCATTCCGTTCTCC |  |  |  |
| Contig-500930 | 500930-F | CCAGACACAGGATTCAGTAACCA | 158 | √ | √ |
|  | 500930-R | CGTAATGCGGTAATCTTTATGTTCT |  |  |  |
| Contig-501250 | 501250-F | CTTGGAGCAGATCAGTTGG | 122 | √ | √ |
|  | 501250-R | TGGTCATTATAGCAATAAAATTTC |  |  |  |
| Contig-501518 | 501518-F | GGTTCAACTCAGGGACAACAC | 141 | √ | √ |
|  | 501518-R | ATATGTCTGAAACCTGAAACACAG |  |  |  |
| Contig-501704 | 501704-F | AATCGGAAAAAAGTTTAGTT | 166 | √ | √ |
|  | 501704-R | GTTGAAACTAGTGAAAACATATC |  |  |  |
| Contig-502042 | 502042-F | TGGACTTGCTTGATGTTCTT | 210 | √ | √ |
|  | 502042-R | AGCAGTGGGTAGTTGGGA |  |  |  |
| Contig-502159 | 502159-F | CATCCTTCATCTGCACATC | 178 | √ | √ |
|  | 502159-R | GTTGATGTTTGATAATTTAATTAG |  |  |  |
| Contig-502369 | 502369-F | GTGAGTGGGCAAAAGGTG | 204 | / | / |
|  | 502369-R | TCTTTAGCCAGGAGTTTCAG |  |  |  |
| Contig-502448 | 502448-F | GATAAGGCATTCTCAAGCAT | 204 | / | / |
|  | 502448-R | ATGCTCCCGCTATCACTC |  |  |  |
| Contig-502878 | 502878-F | GTTCCACAAACCCATCAG | 164 | / | √ |
|  | 502878-R | AAACAATTAAAGACTAATAGCCT |  |  |  |
| Contig-502925 | 502925-F | TGTGTGTCTGTGTTTTGCTAG | 151 | √ | √ |
|  | 502925-R | AAATTTGGTCCGATAATTGA |  |  |  |
| Contig-503239 | 503239-F | GTGATTATTAGGAGACAGGG | 189 | / | / |
|  | 503239-R | CTTTGTCTATGCTTCTCTCTT |  |  |  |
| Contig-503468 | 503468-F | GAATACTTCAGCAAGGCACT | 154 | √ | √ |
|  | 503468-R | CAAAAAACTGCGTTCCAC |  |  |  |
| Contig-503524 | 503524-F | CTTTACTTTCTCTTCAGTGCC | 171 | / | √ |
|  | 503524-R | GACTAAATGTAAATGTTTCAAAAC |  |  |  |
| Contig-503832 | 503832-F | CAGAGATGGCGAGAGATGT | 166 | √ | √ |
|  | 503832-R | GAACGAGAGAATTTTTGAAGAT |  |  |  |
| Contig-503983 | 503983-F | GTTGCCTTGGTAACACTTGAC | 152 | / | / |
|  | 503983-R | GAACCGACAACTGGGGAT |  |  |  |
| Contig-504927 | 504927-F | CACACCTGTCAAGTGATTCTAA | 160 | / | / |
|  | 504927-R | AAACTAAAAAAACGAATCTAACTC |  |  |  |
| Contig-505149 | 505149-F | AGGGGTTTGTTAATAAGAAGA | 183 | √ | √ |
|  | 505149-R | CATATTGGATGTTGTAGGCA |  |  |  |
| Contig-505238 | 505238-F | TCTGGACCAAGTCAGGAACA | 161 | √ | √ |
|  | 505238-R | GGCAGAATGCGTAAGAAGC |  |  |  |
| Contig-505381 | 505381-F | ATCTTTTCTTTGTATGTTTAGTAG | 196 | √ | √ |
|  | 505381-R | ACCACCATCTTTCTCTGAC |  |  |  |
| Contig-505713 | 505713-F | CATGAATCAAAACCTTTTAATAC | 182 | √ | √ |
|  | 505713-R | GTGCCCACATCATTACCAG |  |  |  |
| Contig-505866 | 505866-F | TACTTACTTTGGCACATTGA | 161 | / | / |
|  | 505866-R | CAGGTCAATCTTGGGGA |  |  |  |
| Contig-505934 | 505934-F | AACAAACAAAAAACAAACAC | 206 | / | √ |
|  | 505934-R | TAAATACACTCCCTTAGCAA |  |  |  |
| Contig-9542 | 9542-F | AATACCCTTCAACTGCTGC | 451 | √ | √ |
|  | 9542-R | ACAAGTTCTTTAGCCAGTTATG |  |  |  |
| Contig-53356 | 53356-F | AACAGAACAATGAAGAGCAGG | 473 | √ | √ |
|  | 53356-R | AGCACAGACAGAAAGACCCA |  |  |  |
| Contig-56015 | 56015-F | CTGAACCAGGAGTCACACGA | 358 | √ | √ |
|  | 56015-R | AAACACAAACAGACGAAAGGAT |  |  |  |
| Contig-63607 | 63607-F | ATGAAGAGGATAAAAACAGGAT | 475 | √ | √ |
|  | 63607-R | TTTCCACATCATCTGAGGTT |  |  |  |
| Contig-67123 | 67123-F | CACACACTTATTTTGGGCA | 350 | √ | √ |
|  | 67123-R | TTACACTCTTTTACACAACACAG |  |  |  |
| Contig-67752 | 67752-F | ATTACTAAATCAGCCATCACAG | 399 | / | √ |
|  | 67752-R | ACAACCTCATCCCACAAGA |  |  |  |
| Contig-73826 | 73826-F | TGGCGAGTATTCAAATGTAAG | 510 | √ | √ |
|  | 73826-R | TCACCAGAACAAACAGTAGCA |  |  |  |
| Contig-82018 | 82018-F | TCAGAACAGGCTGGAGTTATTG | 530 | √ | √ |
|  | 82018-R | GTTGGATTGGAAGTTGTAGCAC |  |  |  |
| Contig-92306 | 92306-F | CCACAAAAACACACAGAGTCC | 234 | / | / |
|  | 92306-R | TGTTCCCTGGATTCCTCTCT |  |  |  |
| Contig-506302 | 506302-F | TGGATCAGCCGCTCA | 181 | √ | √ |
|  | 506302-R | AAGAAAAATAAAATTGTATGAAG |  |  |  |
| Contig-506322 | 506322-F | CGACAAACATCCACTAATCTT | 135 | √ | √ |
|  | 506322-R | GGCTGTAACACAACACCTTC |  |  |  |
| Contig-507656 | 507656-F | CTAACACACACATACACGAGG | 179 | / | / |
|  | 507656-R | GCACATTCTTTACAACATTCTAC |  |  |  |
| Contig-507734 | 507734-F | CTCATCATCCCAAACCAGT | 185 | / | / |
|  | 507734-R | CACTGAAACAGAAGGATGGT |  |  |  |
| Contig-508242 | 508242-F | GTAATCCAATCAAGTGTGTAAATAAG | 141 | / | √ |
|  | 508242-R | AGGGGGTGAGCGAAACTG |  |  |  |
| Contig-508554 | 508554-F | ATGTATTTGTTTGTTTCTGTTTT | 146 | / | / |
|  | 508554-R | AGAGAGGAAAAAGAGAGGGA |  |  |  |
| Contig-508606 | 508606-F | CTAATGACTGTGCGGGCTG | 140 | / | / |
|  | 508606-R | ATCTGTGTTTGGGAATCTGACC |  |  |  |
| Contig-508919 | 508919-F | GTCAGCATTGGAACCTAACG | 156 | √ | √ |
|  | 508919-R | CGTCATCTTTTGATCTTAAAGC |  |  |  |
| Contig-509424 | 509424-F | TTATTTTTCTCACTAACATTTT | 170 | / | / |
|  | 509424-R | CTCTTTTTCATTTGTTTTATC |  |  |  |
| Contig-510752 | 510752-F | TATTCTATCATTTATTTTTGGT | 163 | / | / |
|  | 510752-R | GGTCTGTTAGTAAATGAAGGA |  |  |  |
| Contig-511004 | 511004-F | CTGACTGTGACCACCAACT | 151 | √ | √ |
|  | 511004-R | TTAGATTTTAGCTCTTAGGTTTC |  |  |  |
| Contig-511142 | 511142-F | TAATGATACACACACAAAAAGG | 184 | √ | √ |
|  | 511142-R | AGTCATGATCCTAGTTTTTATCTA |  |  |  |
| Contig-511213 | 511213-F | CACCCACATACACACACAAT | 153 | / | / |
|  | 511213-R | GCCACATAGAAGTCCACTTAC |  |  |  |
| Contig-511272 | 511272-F | AATAGGATTTCATAAAAGGACT | 137 | √ | √ |
|  | 511272-R | TGGCCGACATTAGCAAG |  |  |  |
| Contig-511382 | 511382-F | TTCCTTTGGTATAATGTGTG | 184 | √ | √ |
|  | 511382-R | GTAATTCTCCAACACTTAATTT |  |  |  |
| Contig-511514 | 511514-F | GTGTGCGGTGACAGGAA | 147 | / | √ |
|  | 511514-R | ATTTCTGCTAATGGTACCTTC |  |  |  |
| Contig-511834 | 511834-F | AACTCACGAGAAGTCAGGC | 170 | √ | √ |
|  | 511834-R | ACTGAATATAAAATGAGCAAATG |  |  |  |
| Contig-511915 | 511915-F | CTCACTAAACACTTCTCACCA | 152 | / | √ |
|  | 511915-R | CACACATTTTTTGGATTATTAG |  |  |  |
| Contig-512321 | 512321-F | CTGTCACTGGTGCCCCT | 133 | √ | √ |
|  | 512321-R | GGCACACTGTTAGTTCCTCTT |  |  |  |
| Contig-512301 | 512301-F | AGGGAGTAGTCCAGGCTT | 108 | √ | √ |
|  | 512301-R | TTCTATAGTTTACATTTCAAGTCTAC |  |  |  |
| Contig-512381 | 512381-F | CCCTGGAAATGATTGAGA | 193 | √ | √ |
|  | 512381-R | CGAACTAAGAGGAACTACTAAGA |  |  |  |
| Contig-512633 | 512633-F | GAAAAGCGCGAAATTGT | 148 | √ | √ |
|  | 512633-R | AAATAAACCAAAACCTCATCA |  |  |  |
| Contig-513438 | 513438-F | ATGAAAGCAGGTTGAAAGTT | 148 | / | / |
|  | 513438-R | TGTTTAAATAATGTTTAGATACCAA |  |  |  |
| Contig-514819 | 514819-F | GAAGGAGAGAACAGGCAGG | 165 | √ | √ |
|  | 514819-R | GAAACTGTCGGTTACACGGA |  |  |  |
| Contig-515089 | 515089-F | AACTTTACTGTCTGCTCTGCC | 121 | / | / |
|  | 515089-R | TGTTTTTTATTACTCCTGGTGC |  |  |  |

^a^ represented that PCR product was obtained

^b^ represented that no band was obtained in PCR amplification

^c^ represented that this pair of primers produced different bands in F-Mix and M-Mix DNA

^d^ Note: Because of the economic value of the sex-specific molecular markers in the development of all-male breeding industries, so the sequences of the primers used in this report are not public. Please accept our apologies.
